# Cytochrome P450 metabolism mediates low‐temperature resistance in pinewood nematode

**DOI:** 10.1002/2211-5463.12871

**Published:** 2020-05-22

**Authors:** Bowen Wang, Xin Hao, Jiayao Xu, Buyong Wang, Wei Ma, Xuefeng Liu, Ling Ma

**Affiliations:** ^1^ College of Forestry Northeast Forestry University Harbin China; ^2^ College of Agricultural and Biological Engineering Heze University Heze China; ^3^ Heilongjiang University of Chinese Medicine Harbin China; ^4^ Heilongjiang Forest Protection Technology Innovation Center Northeast Forestry University Harbin China

**Keywords:** *Bursaphelenchus xylophilus*, cytochrome P450, low temperature, pinewood nematode

## Abstract

Pinewood nematode (PWN; *Bursaphelenchus xylophilus*) is a devastating invasive species that is expanding into colder regions. Here, we investigated the molecular mechanisms underlying low‐temperature resistance of PWN. We identified differentially expressed genes enriched under low temperature in previously published transcriptome data using the Kyoto Encyclopedia of Genes and Genomes. Quantitative real‐time PCR was used to further validate the transcript level changes of three known cytochrome P450 genes under low temperature. RNA interference was used to validate the low‐temperature resistance function of three cytochrome P450 genes from PWN. We report that differentially expressed genes were significantly enriched in two cytochrome P450‐related pathways under low‐temperature treatment. Heatmap visualization of transcript levels of cytochrome P450‐related genes revealed widely different transcript patterns between PWNs treated under low and regular temperatures. Transcript levels of three cytochrome P450 genes from PWNs were elevated at low temperature, and knockdown of these genes decreased the survival rates of PWNs under low temperature. In summary, these findings suggest that cytochrome P450 metabolism plays a critical role in the low‐temperature resistance mechanism of PWN.

## Graphical abstract

Cytochrome P450 metabolism plays a critical role in the low‐temperature resistance mechanism of pinewood nematode (PWN). Transcript levels of three cytochrome P450 genes from PWN were significantly elevated under low temperature. RNA interference of three cytochrome P450 genes from PWN decreased the survival rates of PWN under low temperature.

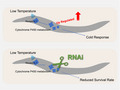

**Abbreviations**
ddH_2_O: double‐distilled water
DEG: differentially expressed gene
FITC: fluorescein isothiocyanate
KEGG: Kyoto Encyclopedia of Genes and Genomes
PWN: pinewood nematode
qRT*‐*PCR: quantitative real‐time PCR
RNAi: RNA interference
SD: standard deviation

Pinewood nematode (PWN;* Bursaphelenchus xylophilus*) is one of the most dangerous plant parasite nematodes that cause devastating pine wilt diseases to the pine trees in Asia, Europe and North America [1, 2]. Although many efforts have been achieved in the prevention of PWNs, the infestation area of PWNs, in all probability, will continue expanding to colder regions of Asia and Europe [3, 4, 5, 6]. For many invasive pests such as PWN, their ability to adapt to different ecological niches is an important determinant of their invasion to new regions [7]. Winter survival ability of PWN in new invasion areas is closely related to the alteration of population structure [8]. In the autumn, adult PWNs stop their growth and gradually enter winter diapause in response to the decreased environmental temperature. At the same time, also stimulated by the decreased temperature, the second‐stage propagative juveniles gradually develop into specialized third‐stage dauer larva (DL_3_). When the following spring comes, the fourth‐stage dauer larva, developed from specialized third‐stage dauer larva, is carried from one pine tree to another by the longhorn beetle, *Monochamus alternatus* [9, 10, 11]. Therefore, the ability of low‐temperature resistance is crucial for not only the survival but also the spread of this devastating pest.

In previous research, our results showed that PWNs revealed a significantly extended life span under 5 °C low‐temperature treatment, compared with 25 °C regular‐temperature‐treated PWNs [12]. Afterward, we also studied the low‐temperature response pattern of PWNs with transcriptome analysis [13]. The study revealed that a large proportion of different expression genes were certain stress‐related genes, including cytochrome P450 genes after treatment with low temperature. This indicated that cytochrome P450 may have potential roles in the low‐temperature responding process of PWN.

Cytochrome P450 is one of the most versatile enzymes in nature. It not only plays a crucial role in many biological functions, such as growth [14, 15], nutrition [16], development [17, 18] and detoxification [19, 20, 21], but also is involved in low‐temperature responding processes of many organisms, including insects [22, 23, 24], plants [25, 26, 27], mammals [28, 29, 30] and nematodes [31]. It has been reported that three pathogenesis‐related cytochrome P450 genes in PWN, *BxCYP33C9*, *BxCYP33C4* and *BxCYP33D3*, are closely related with vitality, dispersal ability, reproduction, pathogenicity, pesticide metabolism [32] and terpene metabolism [33] processes in PWN. However, there are fewer reports on whether cytochrome P450 of PWN is related to low‐temperature response or resistance processes. Therefore, in this study, we performed low‐temperature response pattern research and low‐temperature resistance functional validation of three existing cytochrome P450 genes that were previously cloned by Xu *et al*. [32]. These insights into the low‐temperature resistance function of three cytochrome P450 genes in PWN further illustrated versatile roles of PWN cytochrome P450.

## Results

### Significant enrichment of P450‐related pathways at low temperature in PWN

**Fig. 1:**
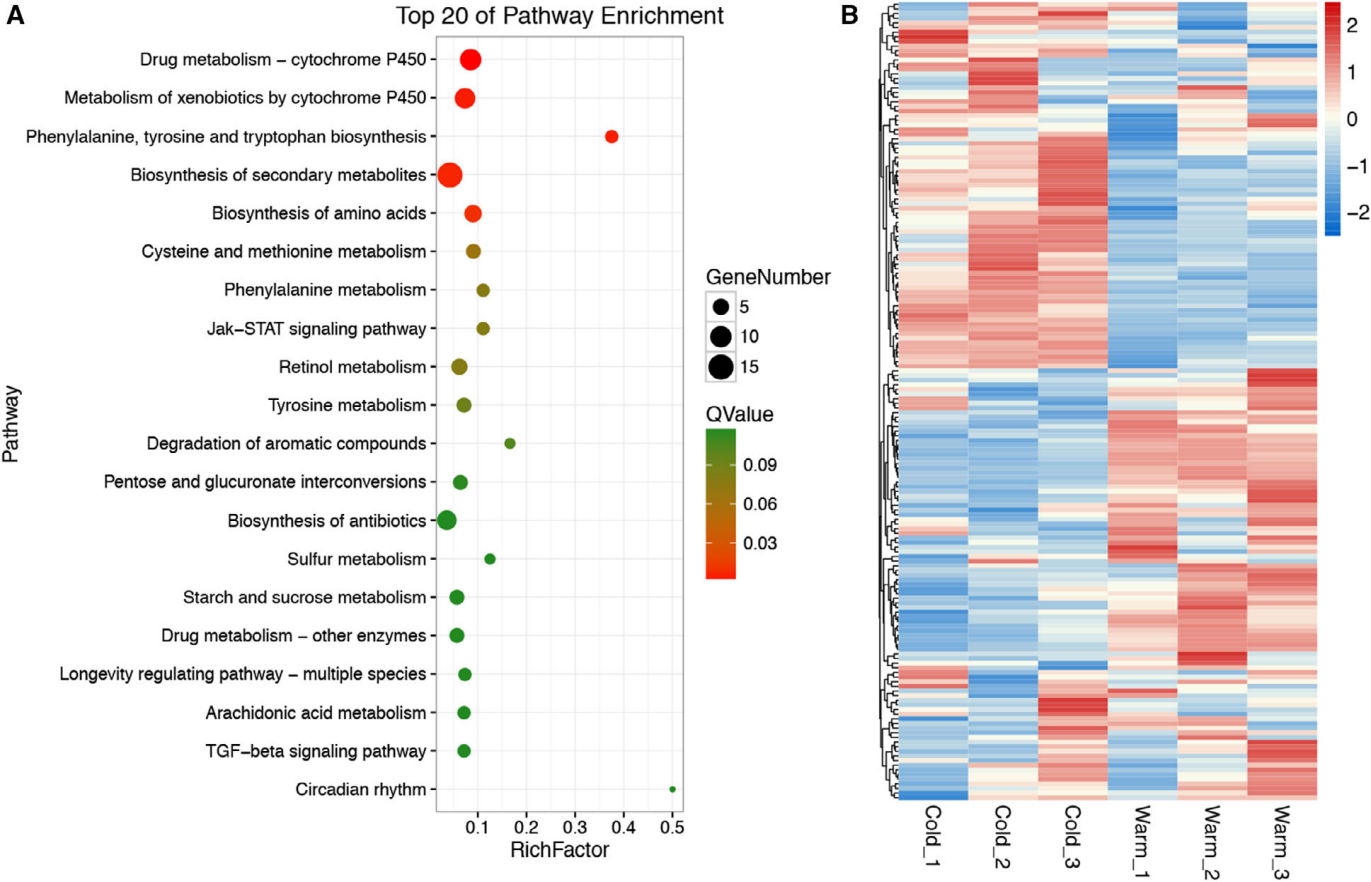
Pathway enrichment and expression analysis revealed a potential role of P450 metabolism in low‐temperature resistance mechanism of PWN. (A) Top 20 of pathway enrichment of DEGs in low‐temperature‐treated PWNs. (B) Heatmap of P450‐related genes transcription under warm‐temperature and low‐temperature treatment.

To analyze the pathway enrichment conditions of low‐temperature‐treated (5 °C) nematodes, we performed pathway enrichment analysis with previously published low‐temperature‐treated PWN differentially expressed genes (DEGs) data. The results showed significant enrichment of DEGs in several metabolism pathways (Fig. 1A; Tables 1 and S1). Two cytochrome P450 metabolism‐related pathways, including drug metabolism–cytochrome P450 pathway and metabolism of xenobiotics by cytochrome P450 pathway, were the top two significantly enriched pathways among all DEGs.

**Table 1:** Top 10 significantly enriched pathways in low‐temperature‐treated PWNs.

No.	Pathway	*P*‐value	*Q*‐value	Pathway ID
1	Drug metabolism—cytochrome P450	0.000015	0.001069	ko00982
2	Metabolism of xenobiotics by cytochrome P450	0.000127	0.004521	ko00980
3	Phenylalanine, tyrosine and tryptophan biosynthesis	0.000225	0.005336	ko00400
4	Biosynthesis of secondary metabolites	0.000375	0.006665	ko01110
5	Biosynthesis of amino acids	0.000708	0.010053	ko01230
6	Cysteine and methionine metabolism	0.005547	0.065639	ko00270
7	Phenylalanine metabolism	0.009429	0.079762	ko00360
8	Jak–STAT signaling pathway	0.009429	0.079762	ko04630
9	Retinol metabolism	0.010111	0.079762	ko00830
10	Tyrosine metabolism	0.012969	0.092078	ko00350

We then performed transcript analysis toward all the cytochrome P450‐related genes, including genes within the top two DEGs enriched cytochrome P450 pathways and genes that have a description as cytochrome P450. The results illustrated that the transcript patterns of cytochrome P450‐related genes were widely different between low‐temperature‐ and regular‐temperature‐treated (25 °C) PWNs (Fig. 1B; Table S2).

### Transcript levels of three P450 genes at low temperature

**Fig. 2:**
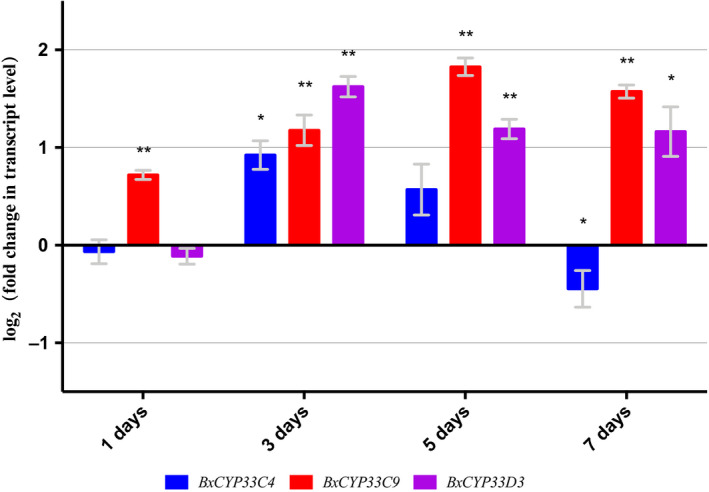
Transcript level analysis of three P450 genes from PWNs under low temperature. Data are mean values ± standard deviation (SD) of different repetitions; *n* = 3. Asterisks indicate statistically significant differences (*P < 0.05, **P < 0.01, Student’s *t*‐test) found between low‐temperature‐ and regular‐temperature‐treated nematodes.

Transcriptome‐based analysis illustrated the potential role of P450 metabolism in the low‐temperature resistance mechanism of PWN. Therefore, we then performed transcript levels analysis of three existing P450 genes, *BxCYP33C9*, *BxCYP33C4* and *BxCYP33D3*, under low temperature with quantitative real‐time PCR (qRT‐PCR) over 7 days. Transcript level changes were calculated between low‐temperature‐ and regular‐temperature‐treated PWNs. The results revealed that three P450 genes of PWN showed an overall higher transcript level at low temperature than regular temperature over 7 days (Fig. 2).

### RNA interference efficiency detection

**Fig. 3:**
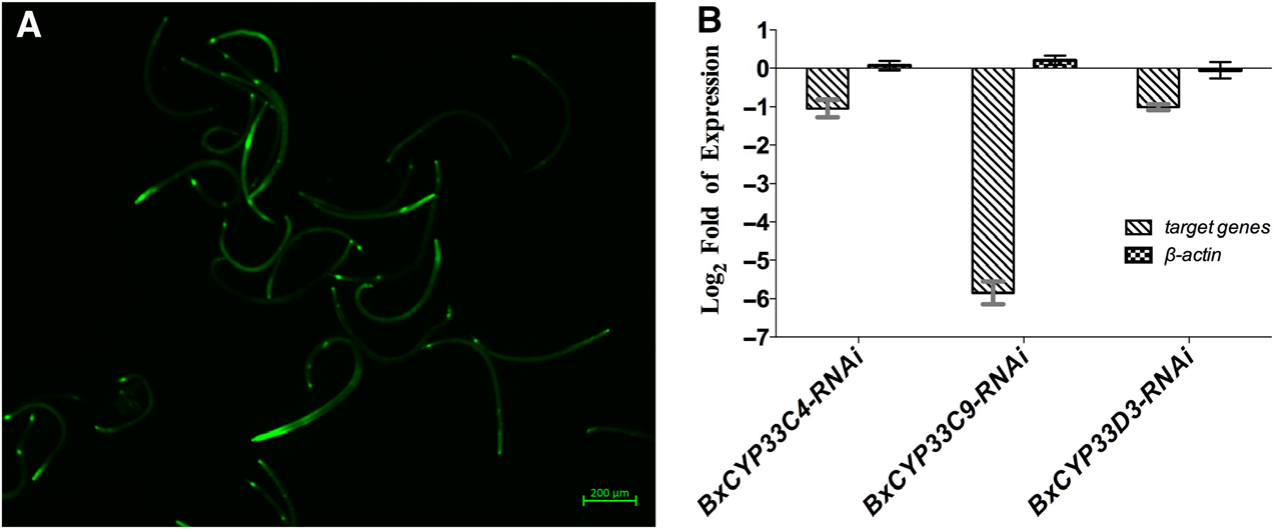
RNAi efficiency detection of three P450 genes from PWNs. (A). FITC‐treated PWN revealed green fluorescent signal under ultraviolet light. (B). Relative transcript level changes between dsRNA‐treated and dsRNA‐free (CK) nematodes. Three P450 genes were significantly silenced after dsRNA treatment, whereas dsRNA had no obvious effect on the transcript level of nontarget *β‐actin*. Scale bar: 200 μm. Data are mean values ± SD of different repetitions; *n* = 3.

To validate the low‐temperature resistance function of three P450 genes from PWN, we then performed RNA interference (RNAi) assay. To examine the efficiency of three PWN P450 genes RNAi, we used qRT‐PCR to detect the transcript level of three PWN P450 genes between control check (CK, dsRNA‐free) and dsRNA‐treated groups. Also, we used fluorescein isothiocyanate (FITC) imaging to indicate the absorption of dsRNA. The results showed that green fluorescence can be detected in the FITC‐treated nematodes under ultraviolet light (Fig. 3A), which indicated that the dsRNA can be absorbed by nematodes with a soaking method. The dsRNA‐treated nematodes showed significantly lower transcript level compared with the CK nematode (Fig. 3B). Silencing rates of *BxCYP33C9*, *BxCYP33C4* and *BxCYP33D3* are 0.78%, 48.41% and 49.65%, respectively. On the contrary, the transcript level of another nontarget internal control gene *β‐actin* did not change after treatment by the dsRNA of three P450 genes (Fig. 3B). These results indicated that the RNAi of these three P450 genes was potent and specific in this experiment.

### Decreased low‐temperature‐resistant ability of PWN after RNAi

**Fig. 4:**
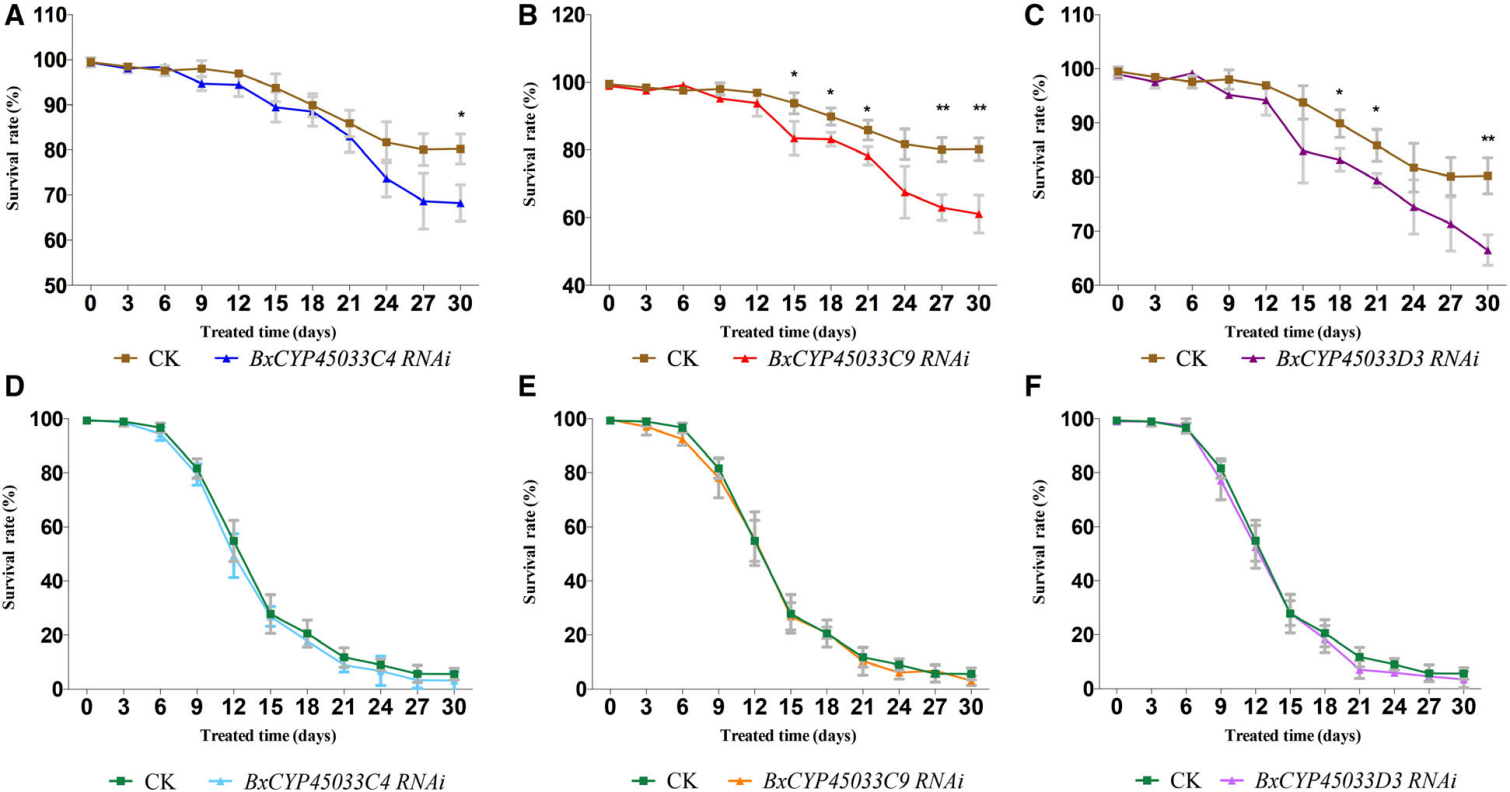
Functional validation of three PWN P450 genes in low‐temperature resistance by RNAi. (A) Survival rates calculation between *BxCYP33C4* dsRNA‐treated and dsRNA‐free (CK) nematodes under low temperature. (B) Survival rates calculation between *BxCYP33C9* dsRNA‐treated and dsRNA‐free nematodes under low temperature. (C) Survival rates calculation between *BxCYP33D3* dsRNA‐treated and dsRNA‐free nematodes under low temperature. (D) Survival rates calculation between *BxCYP33C4* dsRNA‐treated and dsRNA‐free nematodes under regular temperature. (E) Survival rates calculation between *BxCYP33C9* dsRNA‐treated and dsRNA‐free nematodes under regular temperature. (F) Survival rates calculation between *BxCYP33D3* dsRNA‐treated and dsRNA‐free nematodes under regular temperature. Data are for mean values ± SD of different repetitions; *n* = 3. Asterisks indicate statistically significant differences (*P < 0.05, **P < 0.01, Student’s *t*‐test) found between dsRNA‐treated and CK groups.

For the purpose of analyzing the low‐temperature‐resistant ability of PWN after RNAi, we then calculated survival rates of PWN between CK groups and dsRNA‐treated groups under low temperature every 3 days over 30 days. The results showed that RNAi of *BxCYP33C4*, *BxCYP33C9* and *BxCYP33D3* significantly decreased the survival rate of this nematode under low temperature (Fig. 4A–C), whereas few differences in survival rates between the dsRNA‐treated groups and the CK groups could be detected under regular temperature treatment (Fig. 4D–F). These results of decreased survival rates of dsRNA‐treated nematodes under low‐temperature treatment indicated important roles of P450 genes in low‐temperature resistance of PWN.

## Discussion

At present, some efforts have been made in the mechanism of low‐temperature resistance of PWN. The low‐temperature resistance process of PWN is so far known as widely regulated by complicated physiological, biochemical and molecular processes, which include some specific secondary metabolites [10], fatty acid metabolites [34], cGMP pathway [12] and patched‐related proteins [35]. However, to date, the mechanism of the low‐temperature resistance process of PWN remains elusive. In this article, we illustrated the potential role of P450 metabolism in low‐temperature resistance mechanism of PWN through pathway enrichment of DEGs and expression analysis of cytochrome P450‐related genes. In addition, a positively responding pattern of three known cytochrome P450 genes from PWN was further proved by qRT‐PCR. Cytochrome P450 was widely considered as related to the detoxification [36, 37, 38], development [39, 40, 41] and life span regulation function [42, 43, 44]. Elevated expression levels of the three cytochrome P450 genes would be not only a cellular defense mechanism to avoid PWNs from being damaged by low temperature, but also a cellular signal cue for PWNs to enter diapause stage. Furthermore, RNAi of the three cytochrome P450 genes decreased the survival rates of PWNs under low temperature, which illustrated the low‐temperature resistance function of cytochrome P450 in PWNs.

In total, we not only validated the widely different expression pattern of cytochrome P450‐related genes and positive low‐temperature response pattern of three cytochrome P450 genes, but also monitored significantly decreased survival rates of three cytochrome P450 genes of RNAi‐treated PWN under low temperature. These results provided more insights into the molecular mechanism of low‐temperature resistance in PWN, as well as broadened the versatile role of cytochrome P450 from PWN. All of the findings in this article would be helpful in developing cytochrome P450 genes as a target for the management of this devastating nematode.

## Materials and methods

### KEGG pathway enrichment and heatmap analysis

Gene transcription profiles were available from the publicly available data presented in our previous study [12]. DEGs were identified between 5 °C low‐temperature‐ and 25 °C regular‐temperature‐treated PWN groups after 24‐h treatment. Kyoto Encyclopedia of Genes and Genomes (KEGG) was used to perform the pathway enrichment analysis. The calculated *P*‐values were determined through false discovery rate correction with a threshold ≤0.05. Pathways meeting this condition were defined as significantly enriched pathways in DEGs. A transcriptional heatmap of cytochrome P450‐related genes was visualized by r (Bioconductor, Version 3.6.1; Roswell Park Cancer Institute, Buffalo NY, USA) based on normalized fragments per kilobase of transcript per million mapped reads. Cytochrome P450‐related genes were defined as genes within the top two DEGs enriched cytochrome P450 pathways, and genes have a description as cytochrome P450.

### Nematode culture and isolation

PWMs were kindly provided by the Chinese Academy of Forestry, Beijing, China. Nematodes were then maintained in the Forestry Protection Laboratory of Northeast Forestry University, Harbin, China, for further study. *Botrytis cinerea* was used to feed and propagate nematodes at 25 °C. The isolation of PWNs from potato dextrose agar medium plates was performed with the Baermann funnel method.

### Transcript abundance analysis with qRT‐PCR

The transcript abundance was analyzed by the method described previously [12, 34]. Approximately 80 000 nematodes were mixed evenly in 8 mL distilled water. These nematodes were then separated equally into eight 1.5‐mL centrifuge tubes. Four tubes of nematodes were cultured at 5 °C for 1, 3, 5 and 7 days. The other four tubes of nematodes were cultured at 25 °C for 1, 3, 5 and 7 days. The nematodes of both the dsRNA‐treated and the CK groups treated under different temperatures were all treated under the same nutritional status, which is no food supply. Total RNA of nematodes in these eight tubes was extracted and synthesized into cDNA by the following methods: GoTaq 2‐Step RT‐qPCR System (Promega, Madison, WI, USA) was used to synthesize double‐stranded cDNA. Cycle threshold data were used to calculate the relative transcript level (5 °C‐treated group/25 °C‐treated group). The PCR program used in this analysis was as follows: first step, 95 °C for 2 min; and second step, 95 °C for 15 s and 60 °C for 1 min, in 40 cycles. Primers used in the qRT‐PCR analysis were q‐BxCYP45033C4‐F, q‐BxCYP45033C4‐R, q‐BxCYP45033C9‐F, q‐BxCYP45033C9‐R, q‐BxCYP45033D3‐F, q‐BxCYP45033D3‐R, Bx28s‐F and Bx28s‐R (Table 2). The whole transcript abundance study was analyzed in three replicates as three independent trials. Statistically significant differences in transcript level changes between low‐temperature‐ and regular‐temperature‐treated nematodes were calculated with Student’s *t*‐test.

**Table 2:** Primers used in this study.

Gene names	Primer names	Primer sequences 5'–3'	Reference
*BxCYP45033C4*	q‐BxCYP45033C4‐F	AAGATCGACCGCCAGATG	Xu *et al*. [32]
	q‐BxCYP45033C4‐R	CACCTCCAGCTGCATCCT	
*BxCYP45033C9*	q‐BxCYP45033C9‐F	GCGGTTTGCCATGAGACT	Xu *et al*. [32]
	q‐BxCYP45033C9‐R	AAACGGGTGGGATCGAAT	
*BxCYP45033D3*	q‐BxCYP45033D3‐F	CTGATGGGGCAAAGTTGG	Xu *et al*. [32]
	q‐BxCYP45033D3‐R	GCGGGTCCGAAATGTAGA	
*Bx28S*	Bx28s‐F	GTGCGTATTCAGCCTTCTGG	Wang *et al*. [12]
	Bx28s‐R	AACCGAACACGCGACAATAG	
*β‐actin*	β‐actin‐F	TTGGCTGGCCGTGACTTGAC	Li *et al*. [45]
	β‐actin‐R	GCGGTGGCCATCTCCTGTTC	
*BxCYP45033C4*	i‐BxCYP45033C4‐F	TAATACGACTCACTATAGGGTTGGGAGTGAACGGATGA	Xu *et al*. [32]
	i‐BxCYP45033C4‐R	TAATACGACTCACTATAGGGGATCGCATGACTTCTTGTA	
*BxCYP45033C9*	i‐BxCYP45033C9‐F	TAATACGACTCACTATAGGGGCAGCTATGATTGATGGT	Xu *et al*. [32]
	i‐BxCYP45033C9‐R	TAATACGACTCACTATAGGGCAATGAGAAGTAATGTGGC	
*BxCYP45033D3*	i‐BxCYP45033D3‐F	TAATACGACTCACTATAGGGGCCCGAGATTTACCAAGA	Xu *et al*. [32]
	i‐BxCYP45033D3‐R	TAATACGACTCACTATAGGGAACGCATCAATCAGACACTT	

### Synthesis of dsRNA, FITC treatment and gene silencing rate calculation

We performed an RNAi experiment in mixed‐stage PWNs (male : female : juvenile ratio is approximately 1 : 1 : 2) with a soaking method as described in previous studies [12, 34, 46]. MAXIscript T7/T3 RNA Synthesis Kit (Ambion, Tokyo, Japan) was used to obtain dsRNA with RNAi primers (Table 2). The dsRNA‐treated nematodes were soaked in double‐distilled water (ddH_2_O) with 2 mg·mL^−1^ dsRNA corresponding to *BxCYP45033C4*, *BxCYP45033C9* and *BxCYP45033D3* sequence to uptake the dsRNA. FITC was used to monitor the uptake of the dsRNA by nematode. The final concentration of FITC solution was 1 mg·mL^−1^. The CK nematodes were soaked in ddH_2_O only. All of the nematodes were then treated under intermittent stirring for 24 h at 25 °C. Afterward, the nematodes were washed with ddH_2_O to remove the external FITC and dsRNA. A fluorescence microscope (ZEISS, Jena, Germany) was then used to visualize the uptake of FITC by nematodes. After dsRNA uptake, nematodes were divided into two groups: the first group was used to calculate survival rate under 5 °C and 25 °C environments, and the second one was used to calculate the gene silencing rate with qRT‐PCR. Total RNA was extracted from the dsRNA‐treated nematodes and the CK nematodes after RNAi. qRT‐PCR was then performed as outlined earlier with qRT‐PCR primers of *BxCYP45033C4*, *BxCYP45033C9* and *BxCYP45033D3*, and internal control primers *Bx28S* and *β‐actin* (Table 2). Statistically significant differences in transcript level changes between dsRNA‐treated and CK groups were calculated with Student’s *t*‐test.

### Survival rates calculation of PWN under low temperature after RNAi

The survival rates calculation was also performed and analyzed as described in our previous research [12, 34]. Approximately 100 nematodes with 1 mL ddH_2_O in every 1.5‐mL centrifuge tube were used to calculate survival rates of both dsRNA‐treated and CK nematodes under 5 °C low temperature and 25 °C regular temperature. Both the dsRNA‐treated and the CK group nematodes treated under different temperatures were all also treated under the same nutritional status, which is no food supply, as described earlier in Transcript Abundance Analysis with qRT‐PCR. Then the survival rates of nematodes in each centrifuge tube were recorded every 3 days over 30 days. The survival rates calculation method of each tube was as follows: Number of living nematodes in each centrifuge tube / number of total nematodes in each centrifuge tube × 100%. The whole survival calculation work was performed in triplicate. Statistically significant differences of survival rates of each treatment time point between dsRNA‐treated and CK groups were calculated with Student’s *t*‐test.

## Conflict of interest

The authors declare no conflict of interest.

## Author contributions

BoW (Bowen Wang) and LM conceptualized the study. BoW and XH designed the methodology. BoW provided the software. BoW, XH and JX validated the study. BoW analyzed the study. BoW, XH and JX investigated the study. XH and LM provided the resources. BoW, XH, JX and BuW (Buyong Wang) were involved in data curation. BoW wrote the original draft of the manuscript. WM, XL and LM wrote, reviewed and edited the manuscript. BoW and JX visualized the study. XL and LM supervised the study. WM and LM were involved in project administration. LM, BoW and BuW were involved in funding acquisition.

## Supporting information


**Table S1.** KEGG pathway enrichment.

**Table S2.** Expression and KEGG annotations of P450‐related genes.
